# ER intrabody-mediated inhibition of interferon α secretion by mouse macrophages and dendritic cells

**DOI:** 10.1371/journal.pone.0215062

**Published:** 2019-04-16

**Authors:** Konrad Büssow, Philipp Themann, Sabine Luu, Paul Pentrowski, Claudia Harting, Mira Majewski, Veith Vollmer, Mario Köster, Martina Grashoff, Rainer Zawatzky, Joop Van den Heuvel, Andrea Kröger, Thomas Böldicke

**Affiliations:** 1 Department Structure and Function of Proteins, Helmholtz Centre for Infection Research, Braunschweig, Germany; 2 Department Structure and Function of Proteins, Group Recombinant Protein Expression Helmholtz Centre for Infection Research, Braunschweig, Germany; 3 Group Model Systems for Infection and Immunity, Helmholtz Centre for Infection Research, Braunschweig, Germany; 4 Group Innate Immunity and Infection, Helmholtz Centre for Infection Research, Braunschweig, Germany; 5 Department Virale Transformationsmechanismen, Deutsches Krebsforschungszentrum (DKFZ), Heidelberg, Germany; 6 Institute of Medical Microbiology, Otto-von-Guericke-University, Magdeburg, Germany; CIMA, SPAIN

## Abstract

Interferon α (IFNα) counteracts viral infections by activating various IFNα-stimulated genes (ISGs). These genes encode proteins that block viral transport into the host cell and inhibit viral replication, gene transcription and translation. Due to the existence of 14 different, highly homologous isoforms of mouse IFNα, an IFNα knockout mouse has not yet been established by genetic knockout strategies. An scFv intrabody for holding back IFNα isoforms in the endoplasmic reticulum (ER) and thus counteracting IFNα secretion is reported. The intrabody was constructed from the variable domains of the anti-mouse IFNα rat monoclonal antibody 4EA1 recognizing the 5 isoforms IFNα1, IFNα2, IFNα4, IFNα5, IFNα6.

A soluble form of the intrabody had a K_D_ of 39 nM to IFNα4. It could be demonstrated that the anti-IFNα intrabody inhibits clearly recombinant IFNα4 secretion by HEK293T cells. In addition, the secretion of IFNα4 was effectively inhibited in stably transfected intrabody expressing RAW 264.7 macrophages and dendritic D1 cells. Colocalization of the intrabody with IFNα4 and the ER marker calnexin in HEK293T cells indicated complex formation of intrabody and IFNα4 inside the ER. Intracellular binding of intrabody and antigen was confirmed by co-immunoprecipitation. Complexes of endogenous IFNα and intrabody could be visualized in the ER of Poly (I:C) stimulated RAW 264.7 macrophages and D1 dendritic cells. Infection of macrophages and dendritic cells with the vesicular stomatitis virus VSV-AV2 is attenuated by IFNα and IFNβ. The intrabody increased virus proliferation in RAW 264.7 macrophages and D1 dendritic cells under IFNβ-neutralizing conditions. To analyze if all IFNα isoforms are recognized by the intrabody was not in the focus of this study. Provided that binding of the intrabody to all isoforms was confirmed, the establishment of transgenic intrabody mice would be promising for studying the function of IFNα during viral infection and autoimmune diseases.

## Introduction

Interferons (IFNs) are divided into three multigene families (type I, II and III). The type I interferon family comprises the highest number of members: IFNαs, IFNβ, IFNε, IFNƬ, IFNκ, IFNω, IFNδ and IFNξ respectively [1]. Type I IFNs play a major role in the immune response during acute viral and bacterial infections but also take part in induction of tumor cell death and inhibition of angiogenesis [2–4]. They play a pathogenic role in autoimmune diseases and in chronic infections [5, 6].

The type I family members IFNα and IFNβ are produced by almost all cells after contact with microbial products. Their synthesis is induced after binding of danger signals (PAMPs or DAMPs) to some PRRs, especially TLR 7, 9 and RIG-I-like receptors [4]. Activation of PRRs leads to type I IFN synthesis. Binding of type I IFNs to their receptor (IFNAR) induces multiple downstream signalling pathways leading to activation of a large number of IFN-stimulated genes (ISGs) in infected and neighbouring cells [2, 4]. ISG-encoded proteins inhibit the spread of viruses by inhibition of replication, viral transcription and translation, viral assembly and viral egress [7]. Additionally genes are induced that encode cytokines and chemokines, antibacterial effectors and pro-apoptotic and anti-apoptotic molecules [8].

Type I IFNs are produced by virus infected innate immune cells. They can act on innate immune cells including dendritic cells and macrophages enhancing the antigen-presenting function of these cells. In addition virus-infected macrophages and dendritic cells (DCs), the main producers of type I IFNs, secrete IFNα and IFNβ, which can lead to chemokine production in innate immune cells [9]. IFNα and IFNβ activate immature committed DCs to enhance MHC presentation. In addition type I IFNs enhance the antiviral function of adaptive immune cells by promoting CD4^+^ T cells to activate B cells and positively influencing the cytotoxicity of CD8^+^ T cells and NK cells [4].

Dendritic cells can be divided in two main cell types: conventional DCs (cDCs) and plasmacytoid DCs (pDCs). cDCs are specialized in antigen presentation for T-cell activation. The primary function of pDCs is the secretion of high amounts of type I interferons (IFNα and IFNβ) in response to viruses and/or virus-derived nucleic acids. High levels of type I IFN are observed in the beginning of systemic infections such as early murine cytomegalovirus, vesicular stomatitis virus (VSV), lymphocytic choriomeningitis virus and herpes simplex virus type 1. Furthermore, pDCs play a role in human autoimmune diseases such as systemic lupus erythematosus, psoriasis and type I diabetes [10, 11].

The human genome comprises 14 IFNα genes (including one pseudogene) and mouse has 17 IFNα isoforms (including 3 pseudogenes), in contrast to IFNβ that is encoded by a single gene [1, 12–14]. Distinct biological activities of the mouse IFNα isoforms such as induction of IFN-stimulated genes, their expression after viral infection and their anti-viral activity have been studied *in vitro* [15]. The role of IFNα has been studied in infected mice. For example, the reduction of viral titres and of the proportion of infected cells upon treatment of infected mice with different IFNα isoforms have been demonstrated.. In addition, the activation of virus-specific CD4^+^/CD8^+^ T cells and NK cells by IFNα was shown [16–18]. Immunomodulatory effects of IFNα subtypes on NK cells in Hepatitis C infected patients have also been described [19, 20]. However, the *in vivo* functions of IFNα and how they are distinguished from other type I IFN members have remained poorly described. There are no IFNα knockout mice available for analysing the role of IFNα during active and chronic viral infection and in autoimmune diseases *in vivo*. When IFNAR-knockout mice are used, one cannot distinguish between the effects of IFNα and IFNβ.

The function of proteins can be evaluated by knockout techniques such as gene-targeted knockout animals, targeted gene disruption in mammalian cells and knockdown techniques such as siRNA, shRNA, miRNA, CRISPR and TALEN. Complementing these approaches, knockdown of proteins is achieved by dominant negative mutants or inhibitory molecules. In the last years, intracellular antibodies (intrabodies) became increasingly promising due to their high target specificity and the possibility to specifically knockdown proteins with posttranslational modifications. Now it is possible to inhibit virtually any protein of a cell passing the ER or localizing in the nucleus or cytosol using intrabody molecules [21–24]. ER intrabodies are applied as scFv, which are stable in the oxidizing environment of the ER [25]. Cytosolic and nuclear intrabodies are mainly single domain antibodies derived from camels or sharks, which are correctly folded in the reducing milieu of the cytosol or nucleus [23]. scFvs can be constructed from hybridoma clones or by selection from human antibody repertoires by phage display or bacterial, yeast, mammalian cell surface display or ribosome display [26].

ER intrabodies are expressed and retained inside the ER and inhibit protein function by blocking the translocation of the antigen from the ER to the compartment where it normally acts. Intrabodies expressed in the cytosol or nucleus have neutralizing capacity or induce an inactive conformation of the target [21–23]. Even transitory proteins can be inhibited in transgenic ER intrabody mice [27, 28]. Besides intrabodies, small alternative protein scaffolds for targeting intracellular proteins have been developed, such as DARPins, ubiquitin variants, monobodies and affibodies [29]. Some non-antibody binders are already being investigated in clinical studies [30].

Here the intrabody technique was used for knockdown of IFNα secretion in RAW 264.7 macrophages and dendritic D1 cells. To realize this strategy, an ER intrabody was constructed from an anti-mouse IFNα rat hybridoma antibody (4EA1) recognizing five mouse IFNα isoforms. This antibody likely also recognizes other IFNα isoforms, which are highly homologous, but this has not been experimentally confirmed until now. 4EA1 has been used frequenly as a general IFNα antibody, e.g. for measuring total IFNα production by virus infected mouse cells including plasmacytoid dendritic cells [31, 32].

The newly developed intrabody was able to knockdown IFNα in stimulated RAW 264.7 macrophages and dendritic D1 cells. This suggests that specific abrogation of IFNα signalling might also be achieved in intrabody-expressing transgenic mice [27, 28]. This could lead to mouse models for studying the role of IFNα during infections and autoimmune diseases such as systemic lupus erythematosus (SLE) [33].

## Materials and methods

### Cloning of 4EA1 V_H_ and V_L_

For all PCRs, the Expand High Fidelity Plus PCR System (Roche) was used with 2.5 units Expand polymerase per 50 μl reaction, 0.2 mM of each dNTP and 0.4 μM of each primer.

Construction of the anti-IFNα intrabody was performed following the methodology described in [34]. 60 μg RNA (A_260_/A_280_ = 2.1) was isolated from 10^7^ cells of the rat anti-mouse IFNα hybridoma cell line 4EA1 [12] using the Genematrix Universal RNA Purification Kit (Roboklon, Berlin, Germany). First strand cDNA was synthesized with AMV Reverse Transcriptase native, 5x RT buffer (Roboklon, Berlin, Germany) and an oligo(dT)_20_ primer. For cDNA synthesis, 1.6 μg mRNA was combined in a total volume of 34 μl with 2.5 μl of 10 μM oligo(dT)_20_ primer and 10 μl of dNTPs, 5 mM each. For annealing, this mixture A was heated to 65 °C for 5 min and then placed on ice. For mixture B, 10 μl 5x RT buffer, 2.5 μl RNase inhibitor (12.5 U/μl, Roboklon, Berlin, Germany), 2.5 μl DTT, 100 mM were mixed, placed on ice, followed by addition of 1.3 μl AMV reverse transcriptase (10 U/μl). The mixtures A and B were combined, and cDNA was synthesized by incubating for 15 min at 42 °C and 45 min at 50 °C, followed by storage at -20 °C.

The synthesized cDNA was used as template for PCR amplification of immunoglobulin variable domains of the heavy (V_H_) and the light chain (V_L_). 7 combinations of a set of 9 degenerate primers were used in PCR reactions [35, 36] (Table 1). Antisense primer Bi4 was used with sense primers Bi3, Bi3b and Bi3d for amplification of the V_H_ domain, and antisense primer Bi5 was used with sense primers Bi6, bi7, Bi8 and NS21 for the V_L_ domain. For each primer pair, four PCRs with annealing temperatures from 52 to 65 °C were performed. The reaction volume was 40 μl with 0.8 μl cDNA template. In the PCR reaction, 5 min at 94 °C were followed by 30 cycles of 30 s at 94°C, 30 s at the annealing temperature (53–64 °C), and 40 s at 72 °C, followed by 10 min at 72 °C. PCR products were only obtained with primers Bi7 and Bi5 for V_L_ with annealing temperatures of 52–60 °C and with primers Bi3d and Bi4 for V_H_ and an annealing temperature of 53 °C. PCR products were cloned with the TOPO TA cloning kit for sequencing (ThermoFisher Scientific, Darmstadt, Germany), and the inserts of three V_H_ cDNA clones and two V_L_ cDNA clones were sequenced. The three V_H_ clone sequences and the two V_L_ clone sequences were identical.

**Table 1:** PCR primers for immunoglobulin variable region DNA.

Primer	Sequence	Ig domain	orientation
Bi3	5'-GAG GTG AAG CTG CAG GAG TCA GGA CCT AGC CTG GTG-3'	V_H_	sense
Bi3b	5'-AGG TSM AAC TGC AGS AGT CWG G-3'	V_H_	sense
Bi3d	5'-AGG TSC AGC TGC AGS AGT CWG G-3'	V_H_	sense
Bi4	5'-CGA GGG GCC AGT GGA TAG ACA AGC TTG GGT GTC GTT-3'	V_H_	antisense
Bi5	5'-GGG AAG ATG GAT CCA GTT GGT GCA GCA TCA GC-3'	V_L_κ	antisense
Bi6	5'-GGT GAT ATC GTG ATR ACM CAR GAT GAA CTC TC-3'	V_L_κ	sense
Bi7	5'-GGT GAT ATC WTG MTG ACC CAA WCT CCA CTC TC-3'	V_L_κ	sense
Bi8	5'-GGT GAT ATC GTK CTC ACY CAR TCT CCA GCA AT-3'	V_L_κ	sense
NS21	5'-GGT GAY ATY CAR ATG ACN CAR WSN CCN GCN WSN YTN WS-3'	V_L_κ	sense

### Edman sequencing

5 μg purified 4EA1 rat anti-mouse IFNα antibody was prepared in reducing sample buffer and separated by SDS-PAGE. The gel was blotted onto a PVDF membrane. The bands of the antibody’s heavy chain and light chain were visualized by Coomassie staining and cut out for sequencing via N-terminal Edman degradation performed on an Applied Biosystems Procise Protein Sequencer 494C with reagents supplied by the manufacturer (ThermoFisher).

### Construction of intrabodies

**Fig 1:**
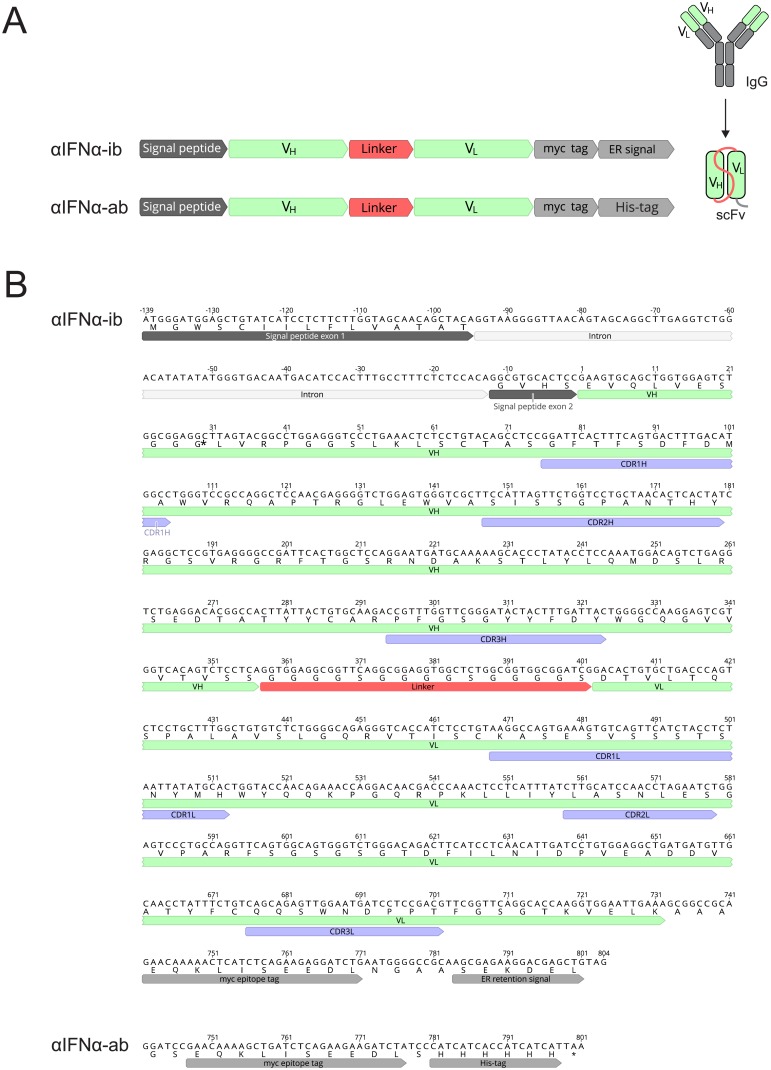
Sequence of the anti-IFNα intrabody αIFNα-ib and soluble scFv αIFNα-ab. A, Scheme of αIFNα-ib and αIFNα-ab. V_H_ and V_L_ were PCR amplified from the cDNA of the complete IgG antibody 4EA1 and a scFv constructed with either a myc tag and ER retention signal or a His-tag. B, Shown are the coding (upper lane) and amino acid sequence (lower lane) including the exons and introns of the signal peptide, the (Gly_4_Ser)_3_ linker, the myc epitope tag and the ER retention signal. The original antibody has a histidine at position 10 of V_H_ (marked with an asterisk). The scFv αIFNα-ab sequence contains a His_6_-tag instead of the ER retention signal. Complementary determining regions (CDRs) are shown as blue arrows.

Intrabodies were constructed from the V_H_ and V_L_ sequences of the complete IgG 4EA1 (Fig 1A). Using assembly PCR [37], V_H_ and V_L_ were linked by the protein sequence (GGGGS)_3_, and a signal peptide was added. The PCR product, comprising signal peptide, V_H_, (GGGGS)_3_ linker and V_L_, was cloned between the NcoI and NotI sites of the vector pCMV/*myc*/ER, thereby adding a myc tag and SEKDEL endoplasmic reticulum (ER) retention signal. First, three PCR reactions (i-iii) were set up (25 cycles, 60°C annealing temperature). (i) The signal peptide was amplified from pCMV/*myc*/ER with primers CMV-For and scFv-AKG-SP (Table 2). (ii) V_H_ was amplified with primers scFv-AKG-VH-S-G and scFv-AKG-VH-AS. (iii) V_L_ was amplified with primers scFv-AKG-Linker and scFv-AKG-VL-AS. Then, 0.5 μl of PCR products i, ii, iii were added to a 100 μl-PCR reaction with primers CMV-For and scFv-AKG-VL-AS (25 cycles). The purified PCR product was digested with NcoI and NotI and ligated to pCMV/*myc*/ER linearized with the same enzymes, creating the plasmid pCMV-αIFNα-ib. The plasmid is available from Addgene.

**Table 2:** Cloning PCR primers.

Primer	Sequence
CMV-for	CGC AAA TGG GCG GTA GGC GTG
scFv-AKG-SP	gcc aga ctc cac cag ctg cac ttc GGA Gtg cac gcc tgt gga gag aaa ggc aaa g
scFV-AKG-VH-S-G	gaa gtg cag ctg gtg gag tct ggc gga ggc tta gta cgg c
scFv-AKG-VH-AS	tga gga gac tgt gac cac gac tcc
scFV-AKG-Linker	gga gtc gtg gtc aca gtc tcc tca GGT GGA GGC GGT TCA GGC GGA GGT GGC TCT GGC GGT GGC GGA TCG gac act gtg ctg acc cag tct cct
scFV-AKG-VL-AS	gtG CGG CCG Ctt tca att cca cct tgg tgc ctg a
IB-LV-for	tcgacggatcgggagatc
IB-LV-rev	actatGTCGACtacagctcgtccttctc
SEW-up	CAC AAC CCC TCA CTC GGC GCG CCA GTC CTC CGA CAG ACT GAG TCG CCC GGG GGG GAT CCA CCG GTC GCC ACC ATG GGT GAC AAT GAC ATC CAC T
SEW-down	TAT CAA GCT TGC ATG CCT GCA GGT CGA CTC TAG AGT CGC GGC CGC TTT ACT TGT ACA GCT CGT CCA
IFNA4-S-Esp3I	CGT CTC Tct ccT GTG ACC TGC CTC ACA CTT ATA AC
IFNA4-AS-Tag	CGT CTC Ttc gaG CTC CTT CTC CTC ACT CAG TCT TG

### Cloning of lentivirus vectors

The intrabody gene was cloned into third generation lentiviral transfer vectors for co-expression of the intrabody with eGFP. The vectors pJSemcveGFP and pHR’SIN-SEW [38] were used for expression with a CMV promoter or an SFFV promoter, respectively. A cassette containing the CMV promoter and αIFNα-ib was PCR amplified from pCMV-αIFNα-ib with primers IB-LV-for/rev (Table 2) and was cloned between the EcoRI and SalI restriction sites of pJSemcveGFP with, upstream of the EMCV IRES and eGFP elements, creating plasmid pLVCMV-αIFNα-ib (S1 Fig). This plasmid was used for expression of αIFNα-ib in RAW264.7 macrophages.

The vector pHR’SIN-SEW contains an eGFP gene activated by an SFFV promoter. The eGFP coding sequence was replaced by a cassette of αIFNα-ib, EMCV IRES and eGFP, which was PCR amplified from pLVCMV-αIFNα-ib with primers SEW-up/down (Table 2). The vector was digested with AscI and SbfI, removing the eGFP ORF, and the cassette was inserted with a QuickFusion kit (Absource, Munich, Germany), creating plasmid pLVSFFV-αIFNα-ib used for expression of αIFNα-ib in dendritic D1 cells.

### IFNα expression plasmid

The coding sequence of mouse IFNα4 (GenBank NM_010504.2) was synthesized (GeneArt, ThermoFisher, Regensburg, Germany). PCR amplification with primers IFNA4-S-Esp3I and IFNA4-AS-Tag was followed by digestion with Esp3I and cloning between the BspI sites of the mammalian expression vector pFlpBtM-II, GenBank KC991095 [39], resulting in the plasmid pFlpBtM-IFNα. The plasmid comprises the IFNα coding sequence without the signal peptide with an N-terminal mouse IgG signal peptide (HVM06_MOUSE) and C-terminal TEV-site, Twin-Strep-tag and His_8_-tag.

### Cells and viruses

HEK293T cells, murine RAW 264.7 macrophages and NIH/3T3 cells were provided by the German strain collection (DSMZ, Braunschweig, Germany).

The dendritic D1 cell line [40] and the X63-GM-CSF cell line were received from Siegfried Weiss, HZI. IEC-Mx2Luc cells were obtained from InSCREENeX [41]. VSV-AV2 was propagated in Vero B4 cells (DSMZ, Braunschweig) as described [42]. The glycosylation deficient cell line HEK293-6E-MGAT1-k.o. was derived from the HEK293-6E cell line [43] by CRISPR/Cas9 deletion of the MGAT1 glycosyltransferase gene [44].

### Cultivation of cells

HEK293T cells, RAW 264.7 cells and Vero 4B cells were cultivated in DMEM (Life Technologies, USA), 10% (v/v) FCS (Life Technologies, USA), 100 μg/ml penicillin G, 100 μg/ml streptomycin (LifeTechnologies, USA) and 1% L-glutamine (Omnilab, Germany).

Dendritic D1 cells were cultivated in the presence of conditioned culture medium. Therefore, 4 x 10^5^ D1 cells/ml IMDM medium (GE Healthcare Life Science, USA) containing 10% FCS, 100 μg/ml penicillin G, 100 μg/ml streptomycin were supplemented with 30% (v/v) of the supernatant of NIH/3T3 cells and 1/20 volume of the supernatant containing 400 ng/ml mouse GM-CSF produced from the X63-GM-CSF cell line and 50 μM ß-mercaptoethanol (Sigma-Aldrich, USA). They were cultivated for 1 week at 37 °C, 5% CO_2_ followed by a new passage. For production of mouse GM-CSF, 5 x 10^5^ X63-GM-CSF cells/ml IMDM supplemented with 5% (v/v) FCS, 1mg/ml G418, 100 μg/ml penicillin G and 100 μg/ml streptomycin were cultivated for 3 days at 37 °C, 5% CO_2_ in 125 ml polycarbonate Erlenmeyer flasks with ventilation membrane caps. After centrifugation, the GM-CSF concentration of the supernatant was determined with a mouse GM-CSF ELISA Ready-SET-Go! Kit (Affymetrix eBioscience, USA) and frozen at -20°C. NIH/3T3-Zellen were cultivated in DMEM + 10% FBS + 1% L-glutamine + 100 μg/ml penicillin + 100 μg/ml streptomycin for 5 days at 37°C, 5% CO_2_ followed by isolation of the supernatant and frozen in 1 ml fractions at -20°C.

For the luciferase assay, 1 ml of 2 x 10^4^ IEC-Mx2Luc cells were grown in a 24-well microtiter plate in muINTEPI medium for 48 h at 37 °C with 5% CO_2_. The IFNα containing supernatants of Poly (I:C) stimulated macrophages and dendritic cells were incubated with the IEC-Mx2Luc cells for 24 h to estimate the IFNα content. Vero 4B cells were cultivated in RPMI 1640, 10% FBS, 1% L-glutamine, 100 μg/ml penicillin and 100 μg/ml streptomycin.

### Production of recombinant lentiviruses and transduction into RAW267.4 cells and dendritic D1 cells

Production of recombinant retroviral viruses was performed in HEK293T cells using Lipofectamine 2000 (ThermoFisher). HEK293T cells were grown on a culture plate of 10 cm in diameter with a cell density of 5 x 10^6^ cells/ml in culture medium without antibiotics. After 24 h, the medium was exchanged with 5 ml fresh medium. Then 2 μg of the packaging plasmid pLP1 (comprising the cDNA of HIV-1 gag and pol, Invitrogen), 4.5 μg of the packaging plasmid pLP2 (comprising the cDNA of HIV-1 rev, Invitrogen) and 2.5 μg of the packing plasmid VSV-G (comprising glycoprotein G of vesicular stomatitis virus, Invitrogen) and 3.0 μg of the lentiviral transfer vector (pLVCMV-αIFNα-ib (S1 Fig), pLVSFFV-αIFNα-ib or the eGFP control vector pHR’SIN-SEW) were diluted in 1.5 ml DMEM medium without FCS and antibiotics. In parallel, 36 μl Lipofectamine 2000 were diluted in 1.5 ml DMEM medium without antibiotics and FCS. After incubation at RT for 5 min, both solutions were combined, shortly shaken and incubated for additional 20 min at room temperature. The DNA-lipofectamin complex containing solution was pipetted dropwise to the cells. After 18 h, the medium was exchanged and cells cultivated for further 72h. Virus-containing supernatants were obtained by centrifugation for 15 min at 3000 rpm and filtered. 1 ml-aliquots were frozen at -80 °C.

For transduction, 264.7 RAW macrophages or dendritic D1 cells were cultivated at a cell density of 10^6^ cells/ml culture medium in a 6-well microtiter plate. After 24 h, the culture medium was exchanged with 800 μl of virus-containing medium supplemented with 8 μg/ml polybren (Sigma-Aldrich, USA) (virus-containing supernatant was diluted 1:2 or 1:4 in medium). 6 h after transduction, 1.2 ml fresh medium was added. The next day, the medium was exchanged and after 24 h, 48h and 96h, the transfection rate determined using flow cytometry by analysing eGFP expression.

### Transient transfection and production of αIFNα-ab and IFNα4

HEK293-6E-MGAT1-k.o. cells were cultivated in FreeStyle F17 medium (ThermoFisher), supplemented with 7.5 mM L-glutamine and 0.1% Pluronic F-68 (AppliChem, Germany), in the presence of 25 μg/mL geneticin. Transient production was performed as described in [45, 46]. The supernatants were harvested at time point 168 h by centrifugation for 30 min at 3000 g and prepared for purification.

### Purification of αIFNα-ab and mouse IFNα4

Both proteins comprising C-terminal poly-histidine tags were purified by immobilized metal ion affinity chromatography (IMAC) using a Profinia protein purification system (Bio-Rad Laboratories, Munich, Germany). Before purification, supernatants were concentrated and dialyzed against 150 mM NaCl, 30 mM HEPES, 30 mM imidazole, 10% (v/v) glycerol, pH 8.0 using a hollow fibre module (cut-off 10 kDa, Spectrum Laboratories, CA). The affinity tag of IFNα, comprising His_8_ and a Twin-Strep-sequence, was cleaved with an excess of TEV-protease (2 mg/ml) overnight at 4°C to prevent binding of αIFNα to the Ni-NTA tips during biolayer interferometry. The protease and uncleaved IFNα was separated from IFNα without tag. This was performed by IMAC using a 1 ml HisTrap FF column (GE Healthcare) and the ÄKTAprime Plus protein purification system.

αIFNα-ab and mouse IFNα4 without tag were concentrated with a Vivaspin concentrator (10,000 MWCO, Sartorius AG, Göttingen, Germany) and further purified by gel exclusion chromatography on a Superdex 75 16/60 column. Proteins were applied to the column after concentration with Vivaspin (Sartorius AG, Germany, MWCO 10,000). Mouse IFNα4 was purified using HEPES buffer (150 mM NaCl, 30 mM HEPES, 10% [v/v] glycerol) and αIFNα-ab was purified using Na-phosphate buffer (150 mM NaCl, 16 mM Na_2_HPO_4_, 84 mM NaH_2_PO_4_ and 10% [v/v] glycerol).

### Determination of the binding kinetics of αIFNα-ab using biolayer interferometry

The dissociation constant of αIFNα-ab was measured by biolayer interferometry with the Octet Red96 system (fortéBio, Menlo Park, CA, USA) using Dip and Read Biosensors (fortéBio) [47]. Kinetic measurements were performed in kinetic buffer (0.2% BSA in PBST, PBST was PBS with 0.05% [v/v] Tween 20). Kinetic buffer without αIFNα-ab was applied to Ni-NTA tips (fortéBio) for 300 sec. Then, αIFNα-ab was coupled in two steps to the Ni-NTA tips. Firstly, the tip was activated for 80 s in 0.1 M EDC, 0.0025 M NHS for covalent coupling and secondly placed for 600 s in a solution of 10 μg/mL αIFNα-ab in kinetic buffer. After covalent binding of αIFNα-ab to the tip, free amino-groups were saturated with 1 M ethanolamine, pH 8.5 for 60 s, followed by equilibration with kinetic buffer for 600 s and recording a baseline for 60 s in wells of a black 96 well microtiter plate (Greiner Bio-One, Austria) to allow subtraction of a baseline drift resulting from unspecific binding of BSA. Tips loaded with αIFNα-ab were then dipped in parallel in 200 μl of increasing concentrations of IFNα in kinetic buffer. The biosensor tips were finally transferred into kinetic buffer to measure the dissociation of αIFNα-ab. Orbital shake speed was 1000 rpm. Fit curves were calculated with the Octet Data Acquisition program 8.2.0.9.

### SDS-PAGE and immunoblot

To demonstrate expression of αIFNα-ib in D1-αIFNα-ib-eGFP cells, 10^6^ cells were incubated in 100 μl Cytobuster protein extraction reagent (Merck) for 5 minutes, centrifuged and heat denatured. 20 μl were analysed by 12% SDS-PAGE and Western blotting. Protein blotting was performed via semi-dry blot onto a PVDF membrane using 48 mM Tris, 39 mM glycine for 30 min at 15 V. After protein transfer, the membrane was washed for 10 min in TBST and blocked for 1 h at room temperature with 3% skimmed milk. Blots were treated for 1 h with mouse anti-myc antibody (9E10, Santa Cruz, 1:2500), washed 3x with TBST, followed by 1 h with goat anti-mouse IgG-Fc alkaline phosphatase labelled antibody (Promega, 1:7500). After washing 3x with TBST and once with AP buffer (100 mM Tris-HCl, pH 9.5, 100 mM NaCl, 5 mM MgCl_2_), blots were developed with BCIP/NPT-substrate (66 μl NBT and 33 μl BCIP in 10 ml AP-buffer, Promega).

### Immunofluorescence

For colocalization analyses, HEK293T cells were grown on sterile coverslips and transiently transfected with αIFNα-ib and mouse IFNα4 expression plasmids. After 48 h, cells were fixed for 15 min with 4% paraformaldehyde at room temperature followed by permeabilisation with 0.1% Triton X-100 in PBS for 15 min. After blocking overnight with 3% BSA in PBS, triple staining of αIFNα-ib, mouse IFNα4 and calnexin was performed with goat anti-c-myc-FITC (1:200 in 3% BSA/PBS, Novus), StrepMAB Classic Chromeo546 (1:500, IBA) and rabbit anti-calnexin (Abcam, 1:75), followed by incubation with a goat anti rabbit Cy5 antibody (1:100, Dianova). Incubation of antibodies was performed over a period of 1 h at room temperature. Between incubation steps, cells were washed once with PBS-0.05% Tween 20. Coverslips were embedded in fluoroshield mounting medium (Abcam) and analysed with a laser scanning confocal microscope (LSM 510 META, Carl Zeiss). For staining of endogenous IFNα, αIFNα-ib and calnexin in RAW 264.7 macrophages and D1 dendritic cells, rabbit anti-calnexin (1:75, Novus Biologicals), rat monoclonal anti-IFNα (F18, Abcam, UK, 1:200), mouse anti-myc (1:100, Santa Cruz SC-40) were applied followed by staining with goat anti-rabbit Cy5 (1:100, Dianova), goat anti-rat Cy3 Jackson/Dianova (1: 200) and goat anti-mouse AMCA (1:200, Abcam, UK) respectively. Alternatively endogenous IFNα was stained with rat anti-IFNα F18 and goat anti-rat Fc Alexa Flour594 labelled antibody (1:200, Abcam, UK) and αIFNα-ib was stained with mouse anti-c-myc antibody and goat anti-mouse IgG (Fc specific) Cy3 labelled antibody.

### Fluorescence microscopy

Confocal analysis was performed with a Zeiss LSM 510 META inverted laser scanning microscope using a Plan-Apochromat 100x oil immersion objective (1.3 numeric aperture). Cells stained with FITC-coupled antibody were excited with an argon laser at 488 nm, and emission was collected using a 505–530 nm bandpass filter. Chromeo 546 and Cy3 were excited using a 543 nm He-Ne laser line and detected using a 560–630 emission filter. Excitation of Cy5-labeled antibody was done with a 633 nm laser line, and emitted light was detected using a 650 nm longpass filter. AMCA was excited by the 364 nm laser line, and emission was collected using a 380–430 nm bandpass filter. Nuclei staining with DAPI was performed for cell detection. Epifluorescence images were acquired with a Zeiss Axiovert 100 microscope using appropriate filter sets for FITC/GFP, Alexa Flour 594 and Cy3 and a Olympus CKX41 microscope using filter sets for FITC and Cy3.

### Co-immunoprecipitation

For co-immunoprecipitation, 10^6^ HEK 293 T cells transiently cotransfected with pCMV-αIFNα-ib and pFlpBtM-IFNα expression plasmids were cultured in a 6-well microtitre plate for 48 h. Cells were harvested by centrifugation at 1000 rpm, and the pellet was incubated with 250 μl NP-40 lysis buffer pH 7.6 (150 mM NaCl, 10 mM Tris-HCl, 1% Nonidet P-40, 5mM EDTA; 1 mM PMSF) for 20 min on ice. 25 μL anti-c-myc agarose beads (anti-c-myc Agarose Affinity Gel; Sigma Aldrich) were washed three times with 500 μL PBST buffer and centrifuged for 5 min at 3000 rpm. 600 μL PBST and 60 μL cell culture lysate were added to the agarose beads and incubated overnight at 4 °C. After incubation, the agarose beads were washed four times with 500 μL PBST buffer. Precipitated complexes were eluted by adding 35 μL Laemmli’s sample buffer and 25 μL PBST and heating for 5 min at 95 °C. The eluted fractions were then analysed by Western blot using a mouse anti-c-myc antibody (9E10, Santa Cruz, 1:2500) for detection the intrabodies or a mouse anti-StrepTag antibody (StrepMAB classic, IBA Lifescience, 1:2000), for detection of IFNα4. As secondary antibody, a goat anti-mouse IgG Fc ab—alkaline phosphatase conjugated (Promega, 1:7500), was applied, and proteins were stained with BCIP/NPT substrate.

### Myxovirus resistance 2 (Mx2) dependent luciferase assay

The amount of secreted IFNα after stimulation of macrophages and dendritic cells was estimated with a Myxovirus resistance 2 (Mx2) promoter-dependent luciferase assay. 1.6 x 10^6^ RAW-αIFNα-ib-eGFP cells or corresponding control cells per well of a 6-well microtiter plate were stimulated by cultivating them for 24 h at 37 °C, 5% CO_2_ in 1 ml DMEM medium containing 10 μl Lipofectamine 2000 and 12.5 μg/ml Poly (I:C) (Invivogen, Toulouse, France). 2 x 10^6^ D1-αIFNα-ib-eGFP cells and control cells were stimulated in 1 ml IMDM medium containing 10 μl Lipofectamine 2000 and 25 μg/ml Poly (I:C) for 24 h. Stimulation experiments were performed in duplicate.

The luciferase assay was performed as follows: IEC-Mx2Luc cells [48] comprising the luciferase reporter gene fused to the Mx2 promoter, inducible by interferon type I and type III, were cultivated in 24 well cell culture microtiter plates with a cell density of 2x10^4^ cells/well in 1 ml medium. After the cells reached 60–70% confluence, 200 μl of 1:5 diluted supernatant of stimulated macrophages or dendritic cells were pipetted to the IEC-Mx2Luc cells and incubated for 24 h at 37 °C, 5% CO_2_. Then the supernatants were discarded, the wells were washed once with 1 ml PBS, and cells were lysed with 125 μl 1 x reporter lysis buffer (RLB, Promega). Plates were stored for at least 3 h at -80°C. For analysing the amount of luciferase, 10 μl cell lysate were added to 100 μl Beetle-Juice (Beetle-Juice Small Kit, PJK GmbH, Kleinblittersdorf, Germany). Luminescence was measured with a luminometer (Lumat LB 9507) for 10 s.

To distinguish between secreted IFNα and IFNβ, the neutralizing αIFNα antibody 4EA1 [12] and a neutralizing αIFNβ antibody, rat hybridoma 7FD3, [12] were applied. 100 μl 4EA1 ascites (1:1000) or 100 μl 7FD3 ascites (1:1000) or 50 μl from both antibodies were added to 100 μl of supernatants of stimulated RAW 264.7 cells or dendritic D1 cells. The supernatants were incubated for 2 h at room temperature and then added to the IEC-Mx2Luc cells. The luciferase assays were performed in duplicate.

### Analysis of eGFP expression in RAW 264.7 and D1 cells by flow cytometry

Lentiviral transduced RAW 264.7 and dendritic D1 cells were sorted by eGFP fluorescence with a FACSAria IIu cell sorter (Becton Dickenson). RAW 264.7 cells were sorted three times and D1 cells twice. Non-transduced RAW 264.7 or D1 cells were applied as negative control. After sorting, cells were cultured in medium containing 10 μg/ml gentamicin. Gentamicin was omitted during following cell passages.

Sorted cells were analysed using an LSR II (Becton Dickenson) or a BD FACSCalibure (Becton Dickenson) flow cytometer (S2 Fig). In preparation for flow cytometry analysis, the cells were harvested, washed once with FACS buffer (PBS containing 2% FBS) and counted. Subsequently they were resuspended in 300 μl FACS buffer containing 1 μg/ml DAPI or 1 μg/ml Pacific Blue and analysed.

### ELISAs

To determine the amount of mouse IFNα in supernatants of transiently transfected HEK293 T cells, 0.1 μg purified hybridoma antibody 4EA1 in 100 μl PBS were coated overnight at 4°C on 96-well MaxiSorb polystyrene assay plates (Nunc). Blocking was performed with 100 μl 3% skimmed milk powder in PBST for 1 h at 37 °C. Serial dilutions of supernatants in PBST-3% skimmed milk were then applied for 2 h at room temperature. After washing 5 times for 3 min with PBST, a mouse anti-Strep-tag antibody conjugated with peroxidase (MAB classic HRP, IBA Lifescience, Göttingen, Germany) was added at 1:2000 dilution for 1 h at room temperature. After washing 5 times for 3 min with PBST, 100 μl TMB-solution (Kem-En-Tec Diagnostics, Taastrup, Denmark) was added to develop the signals. The colour reaction was stopped with 50 μl 2 M H_2_SO_4_ and absorption was read at 450 nm. Assays were performed in duplicate.

For detection of mouse IFNα secreted by stimulated dendritic D1 cells, a mouse IFNα Platinum ELISA detecting mouse IFNα4 and mouse IFNα2 was used (Affymetrix eBioscience, San Diego, CA, USA) according to the supplier’s manual. Assays were performed in duplicate.

### Plaque assay

4 x 10^4^ D1-wt cells, D1-eGFP cells, D1-eGFP- αIFNα-ib cells or RAW-wt cells, RAW-eGFP cells and RAW-αIFNα-ib-eGFP cells in 0.5 ml medium were cultivated overnight in 2 wells each of a 24 well microtiter plate. Then the supernatant was exchanged with 250 μl of VSV-AV2 with a MOI of 0.01, 0.1 and 1 for infection of RAW 264.7 macrophages and a MOI of 0.1, 1 and 10 for infection of dendritic cells in DMEM without FCS and antibiotics. To analyse the effect of the retention of IFNα on virus replication, 50% of wells were filled with the virus dilutions including the neutralizing anti-IFNß antibody 7FD3 (ascites, diluted 1:1000). After 1 h, the supernatants were discarded and 500 μl fresh culture medium with FCS but without antibiotics was added and incubated for 24 h. The supernatants were harvested and serial 1:10 dilutions in 100 μl medium with FCS but without antibiotics were added to Vero 4B cells for 1 h. The Vero 4B cells had been seeded at 2 x 10^4^ cells/well of a 96 microtiter plate at the beginning of the assay. After discarding the supernatants, the cells were overlaid with DMEM containing 50% (v/v) of a methylcellulose solution (20 g/L) and incubated for 3–5 days. Plaques were then visualized by staining of living cells with crystal violet.

## Results

### Generation of an anti-IFNα ER intrabody

To inhibit the secretion of IFNα by an intracellularly produced antibody fragment, an intrabody was designed with the ER retention sequence SEKDEL. The intrabody was made of the variable domain sequences of the anti-mouse IFNα antibody 4EA1 [12]. The variable regions of heavy (V_H_) and light (V_L_) chains of the antibody, encoding the antigen-binding domains, were cloned and sequenced (Genbank MH201402, MH201403). Cloned sequences were compared to N-terminal protein sequences obtained by Edman degradation of the purified 4EA1 antibody chains. The cDNA sequences were matched to the Edman degradation results in the following cloning steps. The antibody’s V_H_ and V_L_ sequences were fused with a linker, generating a single-chain variable fragment (scFv, Fig 1). Two expression plasmids were generated. For intracellular expression of the intrabody, the scFv was fused with the ER retention sequence SEKDEL (αIFNα-ib, Fig 1). For production and purification of the scFv in soluble form for binding studies, the scFv was fused with a His_6_ affinity tag in the second plasmid (αIFNα-ab, Fig 1).

### Binding kinetics of soluble αIFNα-ab by biolayer interferometry

To verify that the anti-IFNα scFv is functional, mouse IFNα4 and the αIFNα-ab scFv were produced and purified and a binding experiment was performed. IFNα4 and the soluble scFv αIFNα-ab were purified from supernatants of transiently transfected HEK293-6E-MGAT1-k.o. cells by nickel affinity chromatography. Subsequent size exclusion chromatography resulted in a major monodisperse elution peak for each protein.

**Fig 2:**
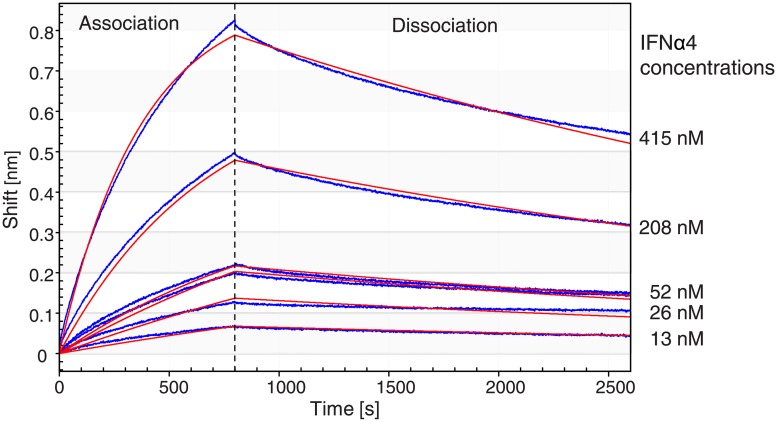
Biolayer interferometry of IFNα4 binding to αIFNα-ab. Real time sensorgrams of αIFNα-ab-loaded Biosensor tips incubated with different concentrations of IFNα4 are shown in blue. Association for 800 s was followed by dissociation of IFNα4 from the tips in buffer for 1800 s. A reference sensorgram measured without IFN4α was subtracted. The binding response is recorded as change in wavelength (shift). A 1:1 interaction model was used to fit the association and dissociation sensorgrams. The fitted curves are shown in red.

The binding of αIFNα-ab to IFNα4 was analysed by Biolayer interferometry using an Octet RED instrument. Binding of molecules to this instrument’s biosensors causes a wavelength shift in the interference pattern, which can be measured in real time. The kinetics of binding and dissociation of IFNα4 to αIFNα-ab immobilized on biosensors was measured at different IFNα4 concentrations. Sensorgrams were recorded consisting of the wavelength shift in the interference pattern over time (Fig 2). The sensorgrams were evaluated assuming a 1:1 interaction and theoretical curves were fitted to the sensorgrams by the instrument software. A reliable curve fit is characterized by the statistical parameters R^2^ (>0.95) and Χ^2^ (<3) [47]. Curve fitting resulted in K_D_ = 38.6 nM for the binding of αIFNα-ab to IFNα4, with R^2^ = 0.998 and Χ^2^ = 1.2563 (Table 3), indicative of moderately strong binding between the scFv and the antigen.

**Table 3:** Constants for the binding of αIFNα-ab to IFNα4.

K_D_ [nM]	k_on_ [M^-1^∙s^-1^]	k_off_ [s^-1^]	R^2^	X^2^
38.6	5.97·10^3^	2.30·10^−4^	0.998	1.2563

R^2^, coefficient of determination, Χ^2^, chi-square test

### Colocalization and binding of intrabody and IFNα4 inside the ER

**Fig 3:**
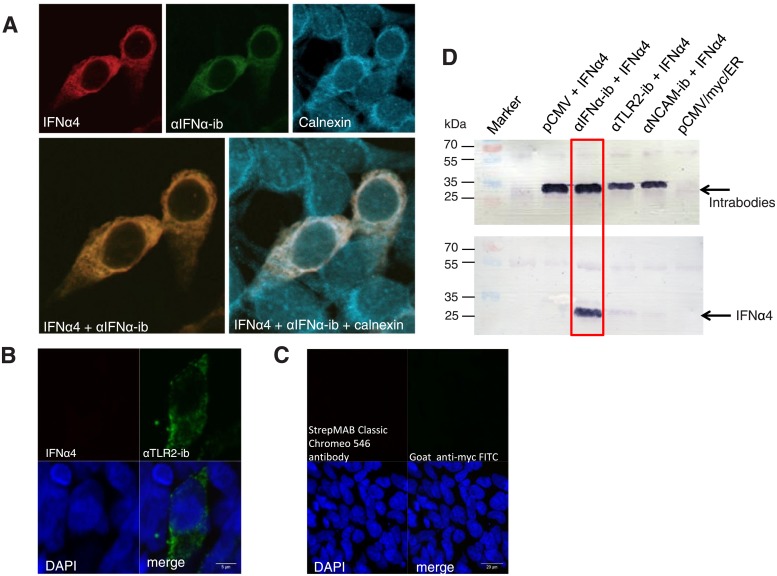
Subcellular colocalization and co-immunoprecipitation of the intrabody and IFNα4 upon transient transfection of HEK293T cells. Immunofluorescence analysis by laser scanning confocal microscopy of fixed and permeabilized HEK293T cells transiently co-transfected with the myc-tagged αIFNα-ib and the Twin-Strep-tagged IFNα4 expression plasmids (**A**) or myc-tagged control intrabody (αTLR2-ib, **B**). Expression of IFNα4 and αIFNα-ib was analysed with StrepMAB Classic Chromeo 546 antibody and goat anti-c-myc-FITC antibody, respectively. Expression of calnexin was visualized with rabbit anti-calnexin and goat anti-rabbit Cy5 antibodies. **C**, As a negative control, cells were co-transfected with the IFNα4 expression plasmid and the empty intrabody vector pCMV/*myc*/ER. Staining as in A resulted in no signal. **D**, Co-immunoprecipitation. HEK293T cells were co-transfected with the αIFNα-ib and IFNα4 plasmids. Negative controls were transfected with IFNα4 and the unrelated, myc-tagged intrabodies αTLR2-ib or αNCAM-ib or the empty vector pCMV/*myc*/ER. 48 h after transfection, co-immunoprecipitation was performed with anti-myc agarose beads, and eluted proteins were visualized by immunoblotting using an anti-c-myc antibody for detection of the intrabodies or an anti-StrepTag antibody for detection of IFNα4.

The intracellular localization of intrabody and IFNα4 was analysed by immunofluorescence of HEK293T cells co-transfected with the IFNα4 expression construct and αIFNα-ib. Cell staining demonstrated colocalization of αIFNα-ib and IFNα4, indicating complex formation of both molecules (Fig 3A). An anti-TLR2 intrabody, αTLR2-ib [49], was used as a negative control and did not have this effect (Fig 3B). Specific intracellular binding of αIFNα-ib and IFNα4 was further demonstrated by co-immunoprecipitation (Fig 3D). Control intrabodies, αTLR2-ib and αNCAM-ib [50], did not precipitate IFNα4, demonstrating specificity of the assay. Another negative control, transfection with only the IFNα4 expression plasmid, did not result in immunoprecipitated IFNα4. The αIFNα-ib:IFNα4 complex colocalized with the ER marker calnexin. Triple staining revealed a lattice structure typical for the ER. These results indicate that the intrabody-antigen complex was localized inside the ER.

### Inhibition of IFNα4 secretion by HEK293T cells after co-transfection of αIFNα-ib and IFNα4 expression plasmids

**Fig 4:**
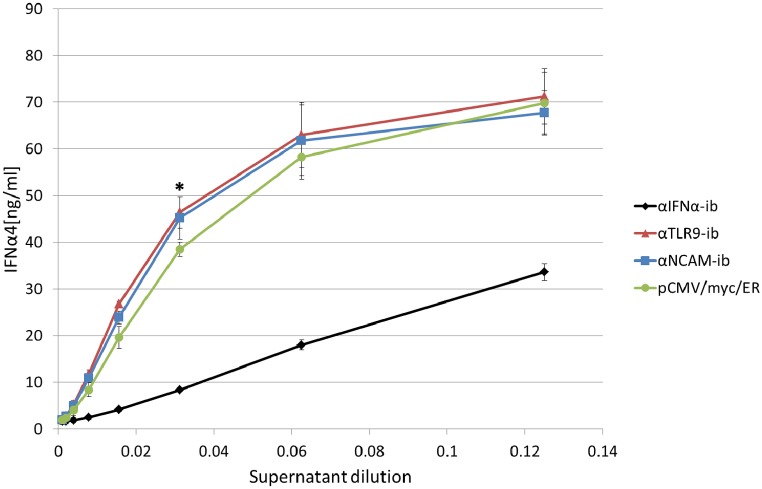
The intrabody inhibits recombinant IFNα4 secretion by co-transfected HEK293T cells. Serial dilutions of supernatants of HEK293T cells transiently co-transfected with Twin-Strep-tagged IFNα4 expression plasmid and αIFNα-ib or control intrabody expression plasmids or empty vector pCMV/*myc*/ER were applied to ELISA plates immobilized with purified anti-IFNα hybridoma antibody 4EA1. Secreted IFNα4 was detected with anti-StrepTag peroxidase-labelled antibody. Bars indicate standard deviations (SD) calculated from two independent experiments. IFNα4 concentration differences between the 1:32 diluted supernatants of the αIFNα-ib plasmid-treated cells (asterisk) and supernatants treated with any of the three other plasmids were statistically significant with *p* < 0.05 (n = 2, one way ANOVA followed by Holm-Sidak test).

αIFNα-ib-mediated inhibition of IFNα4 secretion by HEK293T cells was measured with an IFNα4 ELISA. HEK293T cells were transiently transfected with the IFNα4 expression construct and αIFNα-ib. αTLR9-ib and αNCAM-ib were used as negative controls. Without αIFNα-ib, high amounts of IFNα were secreted and ELISA signals were only linear up to a dilution of 0.03. Only very low amounts of secreted IFNα4 were detectable in the presence of the αIFNα-ib intrabody, whereas expression of the other intrabodies had no effect (Fig 4). Thus it could be demonstrated that IFNα4 secretion was strongly inhibited by the intrabody.

### Characterization of stably transfected intrabody-expressing RAW 264.7 macrophages and dendritic D1 cells

**Fig 5:**
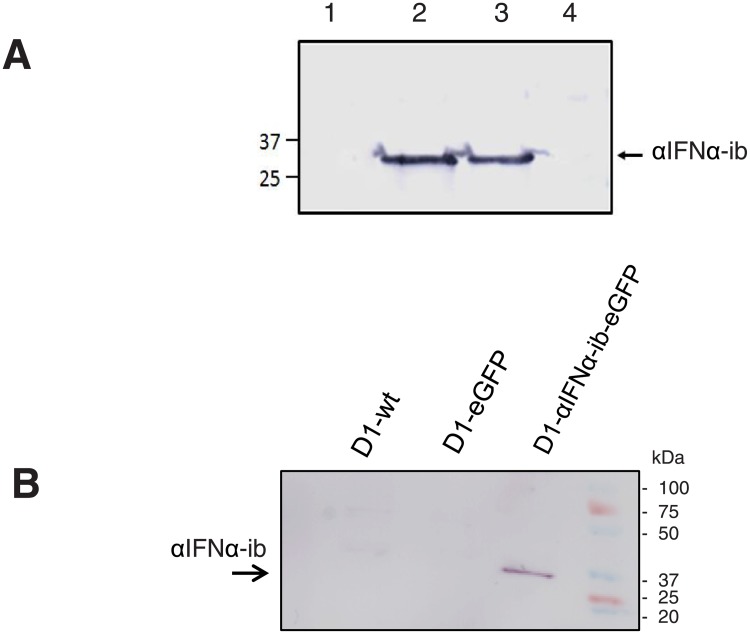
Intrabody expression in stably transfected RAW 264.7 macrophages and dendritic D1 cells. **A**, Expression of αIFNα-ib in RAW 264.7 macrophages analysed by immunoblot after immunoprecipitation. For immunoprecipitation, 10^6^ RAW-αIFNα-ib-eGFP cells were cultured in a 6-well microtitre plate for 48 h. Immunoprecipitation was performed as described in Materials and Methods. The intrabody expressed by RAW-αIFNα-ib-eGFP cells was visualized with mouse anti-myc antibody. 1, Control: RAW-wt cells, 2,3, RAW-αIFNα-ib-eGFP cells, 4, control: RAW-eGFP cells. **B**, Expression of αIFNα-ib in dendritic D1 cells analysed by immunoblot. The intrabody expressed by D1-αIFNα-ib-eGFP cells was visualized with a mouse anti-myc antibody. Negative controls are D1-wt and D1-eGFP cells.

Stably transfected RAW 264.7 macrophages co-expressing αIFNα-ib and eGFP from a CMV promoter were generated by lentiviral transduction and cell sorting (RAW-αIFNα-ib-eGFP). RAW-eGFP control cells, expressing only eGFP, were created in parallel (S2 Fig). Expression of αIFNα-ib was demonstrated by immunoprecipitation. (Fig 5). It is clearly visible that αIFNα-ib is expressed in RAW-αIFNα-ib-eGFP cells.

The D1-αIFNα-ib-eGFP cells were made by transducing D1 dendritic cells with lentiviral particles for co-expression of αIFNα-ib and eGFP, mediated by an SFFV promoter. D1-eGFP control cells expressing only eGFP were also established (S2 Fig). Expression of αIFNα-ib was verified by immunoblot analysis. (Fig 5). The calculated molecular weight of αIFNα-ib, 28 kDa, was confirmed.

**Fig 6:**
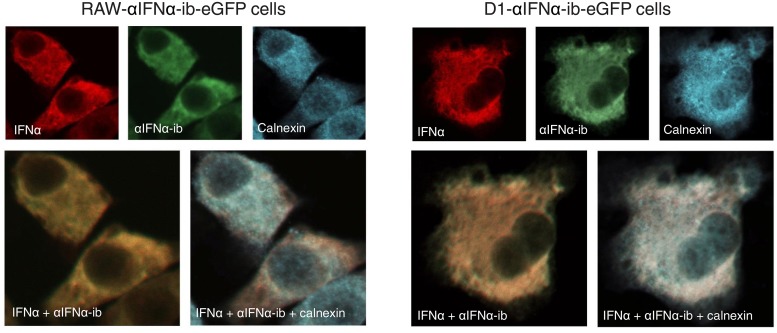
Subcellular colocalization of intrabody and endogenous IFNα in stably transfected macrophages and dendritic cells after stimulation with Poly (I:C). RAW-αIFNα-ib-eGFP macrophages and D1-αIFNα-ib-eGFP dendritic cells were stimulated for 24 hours with Poly (I:C). After fixation and permeabilization of cells, endogenous IFNα, αIFNα-ib and calnexin were stained with rat monoclonal anti-IFNα F18, mouse anti-myc and rabbit anti-calnexin, followed by incubation with goat anti-rat Cy3, goat anti-mouse AMCA and goat anti-rabbit Cy5. Merged images of IFNα and intrabody, and of all three proteins were generated.

To verify the localization of the intrabody-IFNα complex in the ER of stably transfected intrabody expressing immune cells, the stably transfected intrabody-expressing macrophage and dendritic cells were stimulated with Poly (I:C). Intracellular IFNα and intrabody expression were detected by immunofluorescence in both cell populations. Both molecules colocalized with the ER marker calnexin (Fig 6), indicating complex formation of intrabody and IFNα in the ER.

### Intrabody-mediated inhibition of IFNα secretion by RAW 264.7 macrophages and dendritic D1 cells

**Fig 7:**
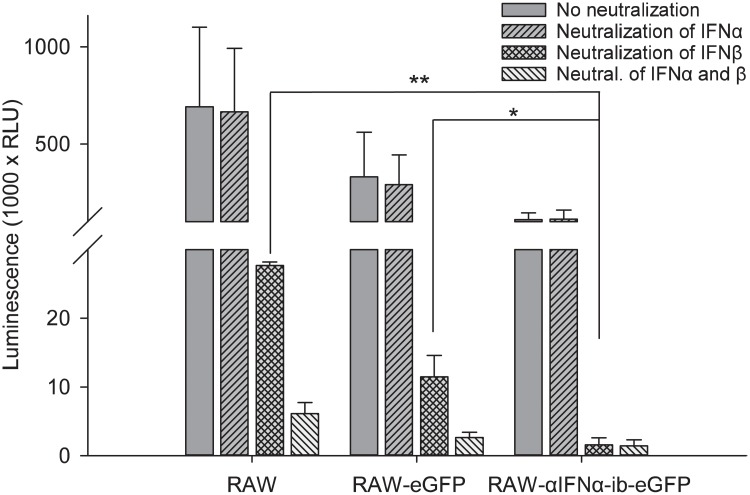
The intrabody inhibits IFNα secretion by RAW 264.7 cells. A, Mx2-dependent luciferase assay. RAW-wt, RAW-eGFP and RAW-αIFNα-ib-eGFP cells were stimulated with Poly (I:C) and the concentration of IFNα/β in the supernatants was quantified by an Mx2-dependent luciferase assay. In addition, IFNα or IFNβ or both interferons were neutralized by addition of the neutralizing antibodies 4EA1 and 7FD3 to the supernatants, as indicated. The diagram shows means ± SD, analysed by two way ANOVA followed by Holm-Sidak test, n = 2, * *p* < 0.05, ** *p* < 0.01. Stimulations assays were performed in duplicate and IFNα/β was measured with triplicate luciferase assays.

The efficiency of the intrabody to inhibit IFNα secretion by RAW 264.7 macrophages was analysed using the RAW-αIFNα-ib-eGFP cells and, as controls, the RAW 264.7 wild type macrophages and the RAW-eGFP cells. The activity of secreted type I IFNs was measured in cell supernatants with an Mx2 promoter-dependent luciferase assay. Supernatants were treated with neutralizing antibodies against IFNα or IFNβ for measuring the IFNs specifically. Neutralization of IFNβ allowed measuring mainly IFNα activity and *vice versa*. Treatment with neutralizing antibodies against IFNα did not reduce IFN activity in the supernatants of wild type and eGFP expressing RAW cells, indicating that IFNβ was the dominant type I IFN (Fig 7). Mx2 promoter activity was lost when both IFN types were neutralized. In contrast, the IFNβ-neutralizing antibody completely abolished reporter gene induction by supernatants of RAW-αIFNα-ib-eGFP cells, indicating that no detectable IFN activity remained. It was concluded that the intrabody mediated pronounced inhibition of IFNα secretion without affecting IFNβ activity.

**Fig 8:**
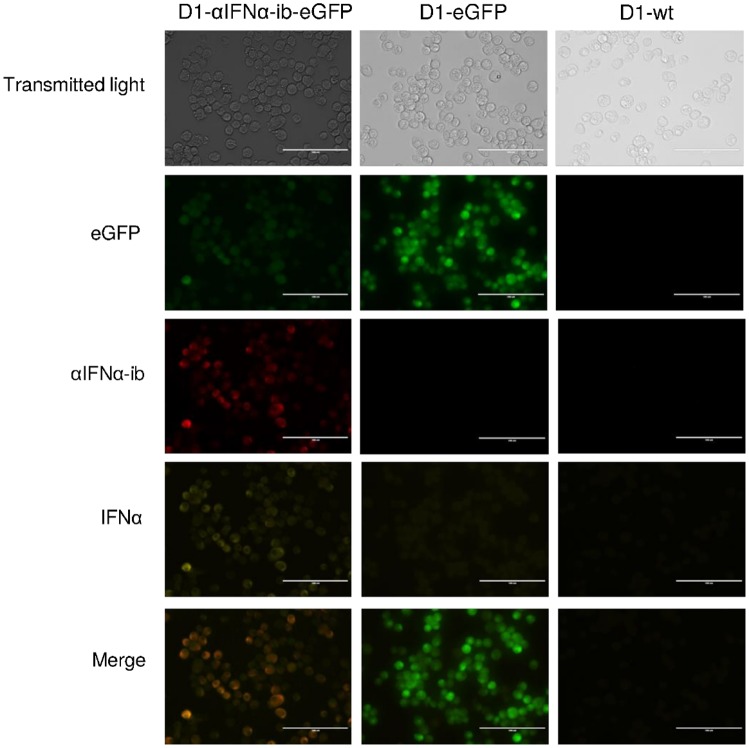
Intrabody-mediated retention of IFNα in Poly (I:C)-stimulated dendritic cells. D1-αIFNα-ib-eGFP cells, control D1-eGFP cells and D1-wt cells were stimulated for 24 h with Poly (I:C), then fixed and permeabilized. Expression of eGFP was detected by its green fluorescence. Endogenous IFNα was stained with rat anti-IFNα F18 and goat anti-rat Fc Alexa Flour594-labelled antibodies and αIFNα-ib with mouse anti-c-myc and goat anti-mouse IgG (Fc specific) Cy3-labelled antibodies.

**Fig 9:**
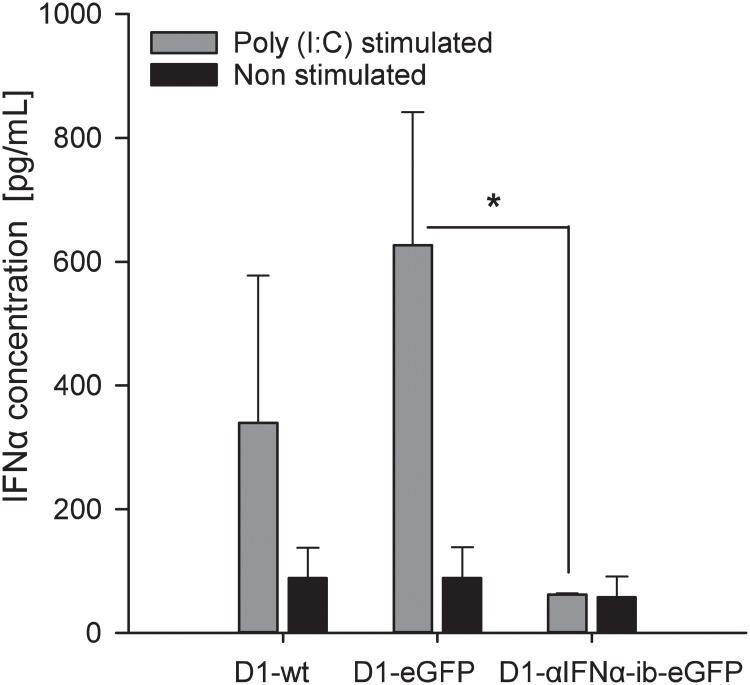
Inhibition of IFNα secretion in Poly (I:C)-stimulated D1-αIFNα-ib-eGFP cells analysed by ELISA. Secreted IFNα was measured in supernatants of Poly (I:C)-stimulated D1-αIFNα-ib-eGFP cells and D1-wt and D1-eGFP control cells using the mouse IFNα Platinum ELISA. Stimulation experiments were performed in duplicate. Data show means ± SD, analysed by two-way ANOVA followed by Holm-Sidak test, * *p* < 0.05.

In a second set of experiments, it was analysed whether αIFNα-ib is able to inhibit secretion of IFNα by dendritic D1 cells. Retention of IFNα by αIFNα-ib was clearly demonstrated by immunofluorescence (Fig 8). Intracellular IFNα was only detectable in Poly (I:C)-stimulated D1-αIFNα-ib-eGFP cells expressing the intrabody. No retained IFNα could be seen in the control D1-eGFP cells or non-transduced D1-wt cells. Retention of IFNα by αIFNα-ib was further confirmed by ELISA measurements of secreted IFNα in D1 cell supernatants (Fig 9). The amount of secreted IFNα in the stimulated D1-αIFNα-ib-eGFP cells was greatly reduced in comparison to the control cells and was similar to the amount obtained from unstimulated D1 cells. In summary, the intrabody effectively inhibited IFNα secretion by Poly (I:C)-stimulated RAW264.7 macrophages and dendritic D1 cells.

### Effect of the intrabody on VSV-AV2 replication in RAW 264.7 and D1 cells

**Fig 10:**
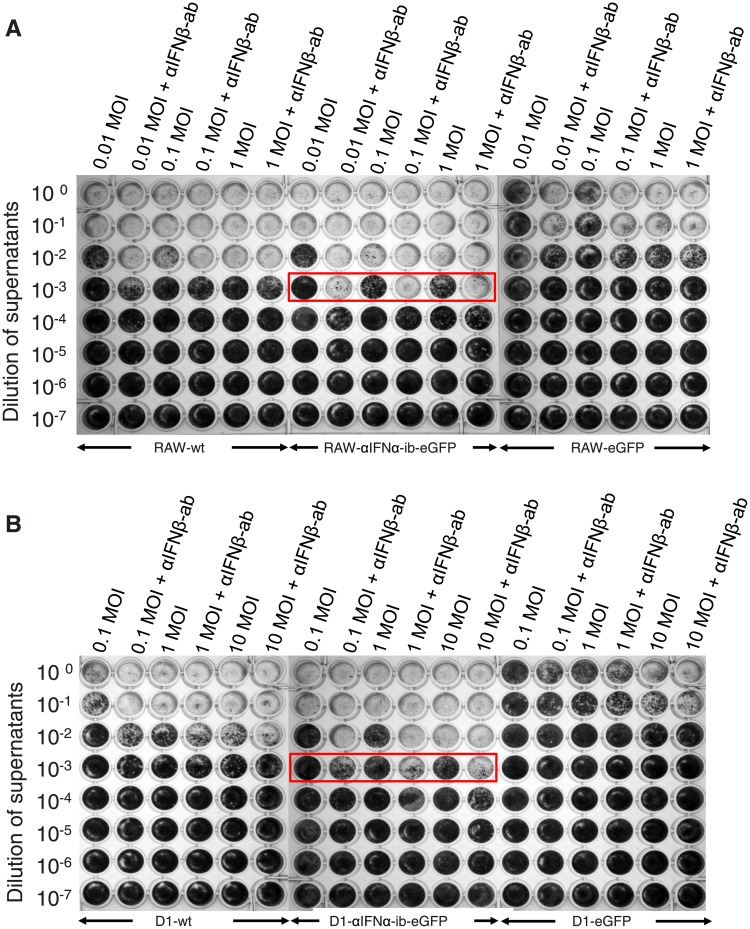
Effect of the intrabody on VSV-AV2 proliferation in RAW 264.7 macrophages and D1 dendritic cells. RAW-αIFNα-ib-eGFP (A) or D1-αIFNα-ib-eGFP cells (B) and control cells were infected with different MOI of VSV-AV2 virus, as indicated. Viral infections were performed with different MOIs in the presence or absence of a neutralizing anti-IFNβ antibody (αIFNβ-ab) for determining the specific effect of IFNα on virus production. The numbers of viruses in serial dilutions of supernatants of the infected cells were estimated by a plaque assay.

The intrabody’s effect on virus replication upon infection of RAW 264.7 macrophages and D1 cells with VSV-AV2 was tested. Upon infection, the VSV mutant AV2 induces type I IFN production and viral titre increases in IFNAR-deficient cells [51]. An IFNβ-neutralizing antibody accordingly lead to an increase in VSV-AV2 titre in wild type and eGFP expressing cells at low MOIs (Fig 10). In contrast, virus replication in cells expressing the αIFNα intrabody and treated with the neutralizing IFNβ antibody was also enhanced at high MOIs (MOI 1 for RAW 264.7 cells and MOI 10 for DCs) and was even stronger at low MOIs compared to control cells (Fig 10, red frames). Together it was concluded that intrabody-mediated inhibition of IFNα secretion removes the virus-inhibitory effect of IFNα.

## Discussion

IFNα activates intracellular anti-viral and anti-bacterial programs and is involved in the development of innate and adaptive immune responses [4]. Furthermore, dysregulation of IFNα responses can contribute to autoimmune diseases and chronic viral infections [5, 6]. IFNαβ has well-documented antiviral effects, and indications for IFNα treatment are chronic viral hepatitis, haematological disorders and solid tumours. Independently of the form (IFNα can be given alone or conjugated to PEG) and administration route, side effects including acute toxicity have been reported [52]. The underlying mechanism of some side effects have been illuminated. For example, Davidson et al. showed that IFNα and β mediate increased mortality in influenza infected mice [53]. They demonstrated that excessive amounts of IFNα and β are secreted by plasmacytoid DCs of influenza infected mice, leading to interaction of the death-inducing receptor DR5 on lung epithelia cells with Trail on inflammatory monocytes, which results in lung tissue damage.

The anti-viral properties of IFNα have been studied *in vitro* and with a few infectious mouse models [15–18]. Nevertheless only little is known about the mechanism of *in vivo* action of IFNα during viral infections, particularly in macrophages and dendritic cells. Gene knockout mice established by gene targeting or CRISPR/CAS technology are valuable for studying the function of proteins *in vivo*. Mice have 17 genes of IFNα isoforms, including 3 pseudogenes. Genetic knockout of all genes would require a large effort. Therefore an IFNα knockout mouse has not been reported yet. The intrabody technology was used here as an alternative to a genetic IFNα knockout. It is the first intrabody inhibiting a secretory antigen in dendritic cells.

The K_D_ of αIFNα-ab to mouse IFNα4 was estimated by biolayer interferometry to 38.6 nM. This value is moderate for an scFv. Interestingly the K_D_ values of an antibody Fab fragment broadly neutralizing human IFNα isoforms were estimated to range from 0.018 nM to 35.4 nM for different IFNα isoforms [54]. The binding affinity of the αIFNα-ab scFv lies between the binding affinities which were estimated for 12 human IFNα subtypes to IFNAR1 and IFNAR2, namely 0.5–5 μM for IFNAR1 and 0.4–5 nM for IFNAR2 [55]. It is known that high antigen affinity is not required for an antibody to function efficiently inside the ER as an ER intrabody [56, 57]. This might be explained by the relatively high concentrations of intrabody and antigen in the ER compartment.

It could be shown by ELISA that secretion of recombinant IFNα4 is inhibited by the αIFNα-ib intrabody in HEK293T cells (Fig 4). Retention of IFNα4 in the ER was based on intracellular binding of both partners, as demonstrated by co-immunoprecipitation (Fig 3). Co-staining of the ER marker calnexin with the intrabody and IFNα4 revealed that the antigen-intrabody complex is localized inside the ER (Fig 6). Similar results had been demonstrated with anti-TLR2, anti-TLR9 and anti-polysialyltransferase intrabodies [34, 49, 50].

Stably transfected cell pools were generated for demonstrating intrabody-mediated inhibition of IFNα secretion by macrophages and dendritic cells. For generation of stably transfected RAW 264.7 macrophages, a lentiviral transfer vector was used for bicistronic expression of the intrabody and eGFP by a CMV promoter. Transduction of dendritic D1 cells with the same vector lead to only 2% eGFP positive cells. One explanation could be that the CMV promoter was inactivated in dendritic D1 cells due to methylation, as demonstrated in some CHO cell lines [58, 59]. For generating the intrabody-producing D1 cells, another vector, based on pHR’SIN-SEW and containing the retroviral spleen focus-forming promoter (SFFV), was constructed. The new vector lead to much better eGFP expression and resulted in 13% eGFP positive D1 cells. Other publications reported 5–20% of GFP expression after retroviral transduction of mouse dendritic cells [60, 61]. In addition to the SFFV promoter, the new vector contains the woodchuck hepatitis post-transcriptional regulatory element (WPRE), which favours gene expression and mRNA stability [62]. This could also explain the vector’s higher efficiency. Interestingly, transduction of D1 cells with the pHR’SIN-SEW control vector resulted in about 34% eGFP positive cells, and these cells expressed higher levels of eGFP (Fig 8). A possible reason is that the distance of the IRES sequence to the first codon of the eGFP cistron was not optimal in the bicistronic construct [63].

The inhibition of IFNα secretion by intrabody-expressing RAW 264.7 macrophages was clearly demonstrated by an Mx2 reporter-dependent luciferase assay (Fig 7). Analysis showed that less type I IFN was found in supernatants of RAW-eGFP cells compared to RAW 264.7 wild type cells. The overexpression of eGFP could have had an influence on cytokine expression. Whether the insertion of small HIV genome elements and the Hepatitis B virus posttranscriptional regulatory element from the lentiviral vector have also an effect on cytokine expression remains unknown. Inhibition of IFNα secretion by intrabody-expressing dendritic D1 cells was also clearly demonstrated (Figs 8 and 9).

At the moment it is not known which IFNα subtypes are secreted after stimulation of RAW 264.7 macrophages and dendritic D1 cells with Poly (I:C) or upon VSV-AV2 infection. Clearly, IFNα secretion is effectively inhibited by the αIFNα-ib intrabody. Preliminary results with VSV-AV2 infected cells showed a significant influence of the intrabody on virus replication by RAW 264.7 macrophages and dendritic D1 cells under IFNß-neutralizing conditions (Fig 10).

In the paper significant inhibition of IFNα secretion by RAW264.7 macrophages and dendritic D1 cells has been demonstrated. However, before establishing a transgenic intrabody mouse, it should be analysed whether the intrabody is also able to efficiently inhibit IFNα secretion in plasmacytoid dendritic cells after viral infection. A further prerequisite for intrabody functionality in a transgenic mouse would be that it recognizes all IFNα isoforms. The 4EA1 antibody was used in several studies for detection of total IFNα [31, 32]. Nevertheless, it would be recommended to clarify this before establishing transgenic intrabody mice.

In summary, the new intrabody can be used *in vitro* for evaluating the effect of IFNα on virus infection in macrophages and dendritic cells. IFNα knockdown in transgenic intrabody-expressing mice could enable studying the role of IFNα in virus infections and in autoimmune diseases, such as SLE [33].

## Supporting information

S1 Fig Retroviral IB vector. (PDF)

S2 Fig FACS Analysis with RAW264.7 macrophages and dendritic D1 cells. (PDF)

S1 Table Raw data files for Figs 4, 7 and 9. (XLSX)
