# Supplementary material for: ER intrabody-mediated inhibition of interferon α secretion by mouse macrophages and dendritic cells
Source: PLoS One. 2019 Apr 16;14(4):e0215062. doi: 10.1371/journal.pone.0215062 (PMC6467385; doi:10.1371/journal.pone.0215062)
Supplement: S1 Fig — (PDF) [file pone.0215062.s001.pdf]

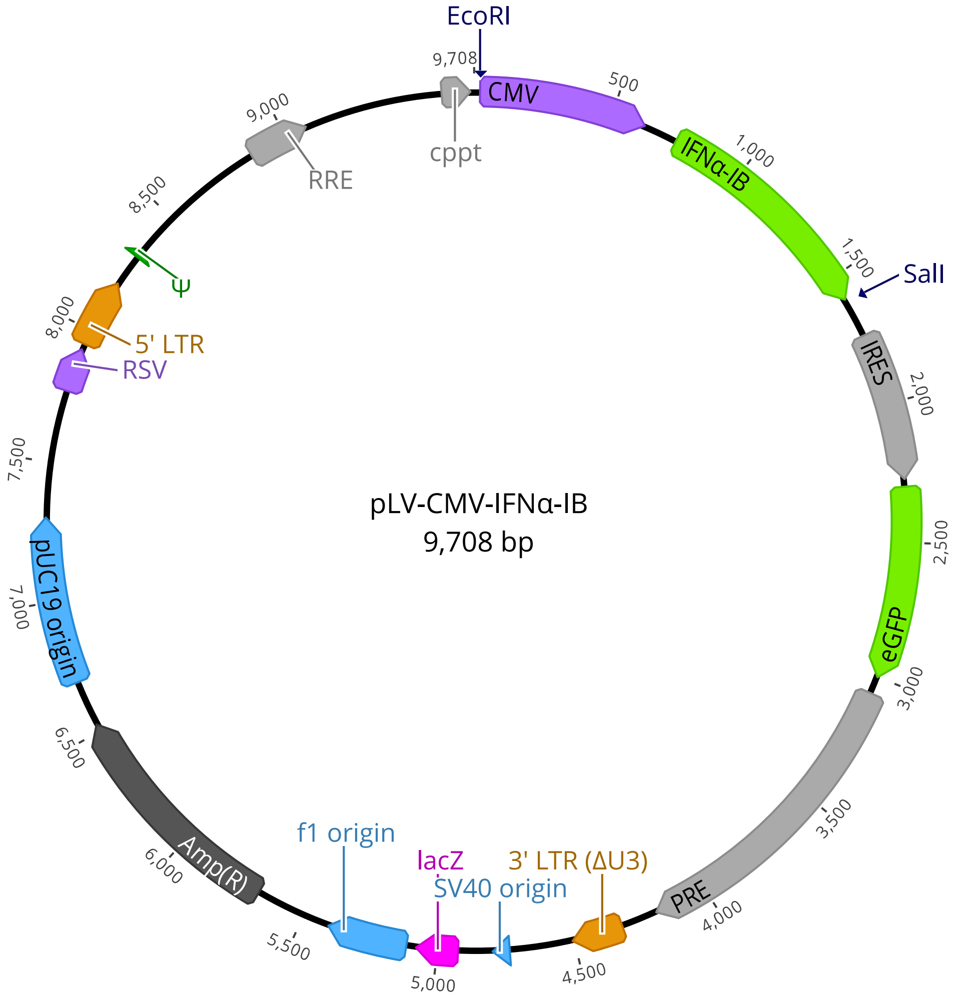

S1 Fig. Third generation lentiviral transfer vector for IFNα-IB co-expressed with eGFP.
HIV: Human immunodeficiency virus 1, RSV: Murine Rous sarcoma virus enhancer/promoter, 5' LTR: 5' long terminal repeat, Ψ: HIV Psi packaging element, RRE: HIV rev response element, cppt: HIV central polypurine tract, IRES: EMCV internal ribosomal entry site, PRE: Hepatitis B virus posttranscriptional regulatory element, CMV: CMV promoter, IFN α-IB: anti-mouse IFNα intrabody, 3'-LTR: HIV 3' self inactivating (ΔU3) long terminal repeat.
