# Supplementary material for: ER intrabody-mediated inhibition of interferon α secretion by mouse macrophages and dendritic cells
Source: PLoS One. 2019 Apr 16;14(4):e0215062. doi: 10.1371/journal.pone.0215062 (PMC6467385; doi:10.1371/journal.pone.0215062)
Supplement: S2 Fig — (PDF) [file pone.0215062.s002.pdf]

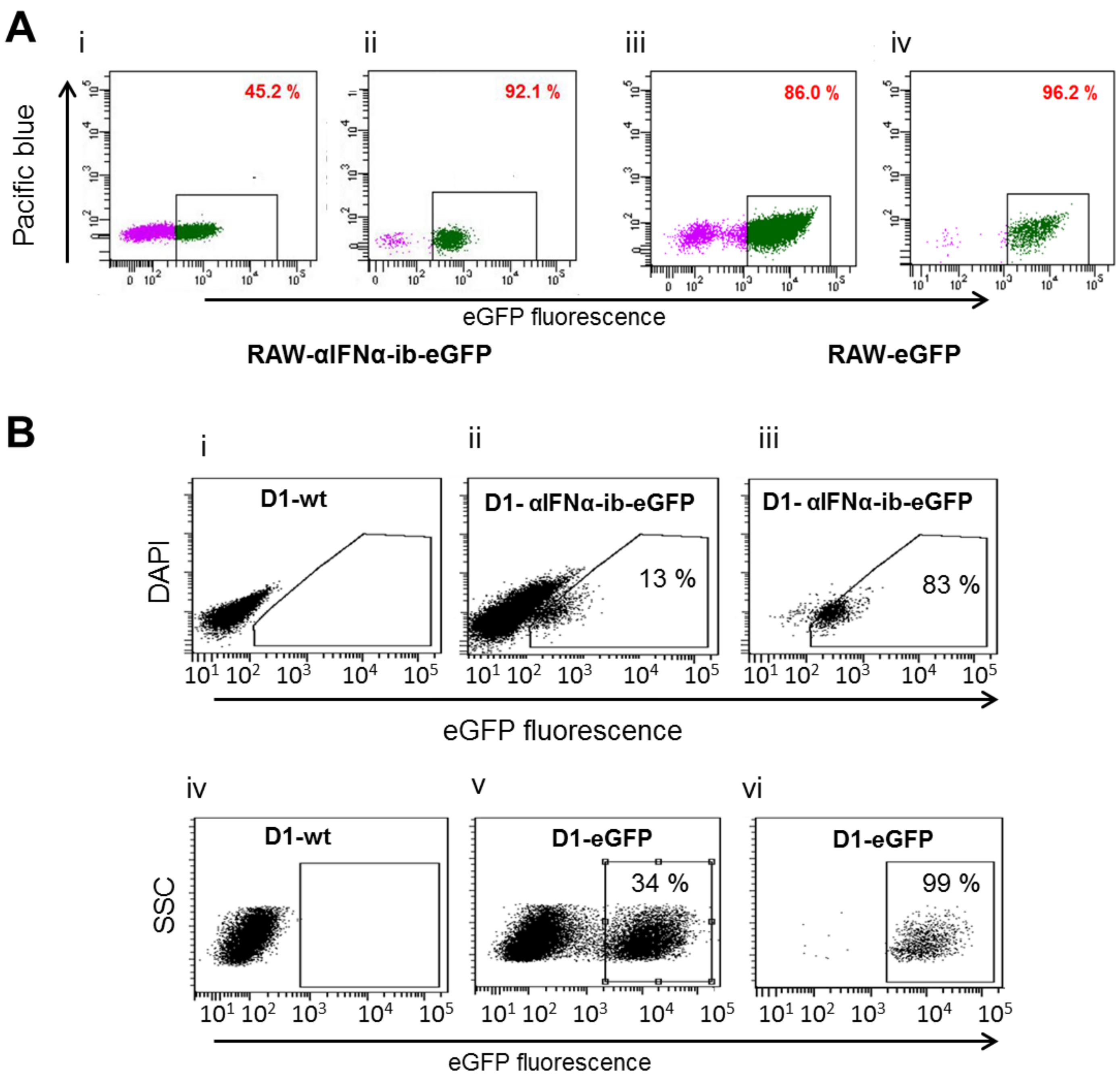

S2 Fig. Analysis of lentivirally transduced RAW 264.7 macrophages and dendritic D1 cells before and after sorting by flow cytometry

A, Upon transduction of RAW 264.7 cells with recombinant lentiviruses generated with transfer vectors pLVCMV-αIFNα-ib or pHR'SIN-SEW, cells were sorted for isolating αIFNα-ib and eGFP-expressing cells (RAW-αIFNα-ib-eGFP; i, ii) or only eGFP-expressing control cells (RAW-eGFP; iii, iv). i and iii show eGFP fluorescence of cells before sorting and ii and iv after three rounds of sorting. Non-transduced RAW 264.7 cells were used as reference. Dead cells were identified by staining with Pacific blue. Shown are living, gated cells. Green and pink dots represent eGFP-positive and negative cells, respectively. B, i-vi, Upon transduction of D1 cells with recombinant lentiviruses generated with transfer vectors pLVSFFV -αIFNα-ib or pHR'SIN-SEW, cells were sorted twice to generate stable eGFP (D1-eGFP) or αIFNα-ib and eGFP (D1-αIFNα-ib-eGFP) expressing cells. Shown are eGFP fluorescence of D1-αIFNα-ib-eGFP cells before (ii) and after sorting (iii). Panel v depicts eGFP fluorescence of D1-eGFP cells before sorting and vi after sorting. Non-transduced D1 wild type cells (D1-wt) are shown as reference (i, iv). eGFP fluorescence of gated living cells is shown. D1-αIFNα-ib-eGFP cells were stained with DAPI to clearly distinguish the eGFP-positive cells from non-transduced cells (ii, iii).
